# A detailed kinetic model of glycolysis in *Plasmodium falciparum*-infected red blood cells for antimalarial drug target identification

**DOI:** 10.1016/j.jbc.2023.105111

**Published:** 2023-07-29

**Authors:** David D. van Niekerk, Francois du Toit, Kathleen Green, Danie Palm, Jacky L. Snoep

**Affiliations:** 1 Department of Biochemistry, Stellenbosch University, Matieland, South Africa; 2 Molecular Cell Biology, Vrije Universiteit, Amsterdam, The Netherlands

**Keywords:** malaria, red blood cell, mathematical modelling, glycolysis, glucose transport, systems biology

## Abstract

Upon infection by the malaria parasite *Plasmodium falciparum*, the glycolytic rate of a red blood cell increases up to 100-fold, possibly contributing to lactic acidosis and hypoglycemia in patients with severe malaria. This dramatic increase in glucose uptake and metabolism was correctly predicted by a newly constructed detailed enzyme kinetic model of glucose metabolism in the trophozoite-infected red blood cell. Subsequently, we expanded the model to simulate an infected red blood cell culture, including the different asexual blood-stage forms of the malaria parasite. The model simulations were in good agreement with experimental data, for which the measured parasitic volume was an important parameter. Upon further analysis of the model, we identified glucose transport as a drug target that would specifically affect infected red blood cells, which was confirmed experimentally with inhibitor titrations. This model can be a first step in constructing a whole-body model for glucose metabolism in malaria patients to evaluate the contribution of the parasite's metabolism to the disease state.

Malaria affected an estimated 241 million people in 2020, up from 227 million in 2019 with the highest impact in the WHO African Region which accounts for 95% of cases. In 2020, malaria deaths were estimated at 627,000. An additional issue of concern is that malarial parasites are gaining resistance to the current artemisinin combination therapies, especially evident in the African region (https://www.who.int/teams/global-malaria-programme/reports/world-malaria-report-2021, accessed November 1, 2022). Of the four *Plasmodium* species that cause malaria, *Plasmodium falciparum* is still the most frequently occurring and the most lethal (https://www.who.int/news/item/03-06-2022-updated-who-recommendations-for-malaria-chemoprevention-and-elimination, accessed November 1, 2022).

The *P. falciparum* malarial parasite is transmitted to a human host by the bite of an infected female *Anopheles* mosquito. After human infection occurs, sporozoites mature in the hepatocytes which release merozoites into the blood circulatory system when rupturing 6 to 7 days later (1). These merozoites invade red blood cells (RBCs) and a repeated cycle of asexual reproduction begins where the parasites start as rings, mature into trophozoites, and finally become multi-nucleated schizonts which release merozoites upon rupture to reinvade other RBCs. It is during this asexual blood stage of malaria that clinical symptoms manifest.

Symptoms that are indicators of severe malaria and a poor prognosis of survival include hypoglycemia and lactic acidosis (2, 3, 4). An altered host glucose metabolism is therefore implicated in the manifestation of these symptoms (5, 6). When infected, a number of mechanisms can contribute to the disruption of the host’s glucose homeostasis stemming from reduced perfusion of capillaries due to the sequestration of infected and rosetting of uninfected RBCs (leading to anaerobic tissue glycolysis and insufficient liver and kidney clearance of lactate) (7, 8); an additional burden on the immune system (9); metabolic effects of inflammatory cytokines (10, 11); and the metabolism of the parasites (9, 10, 12, 13, 14).

In the intraerythrocytic blood phase, the parasite has access to the RBC cytosol, containing the nutrient-rich hemoglobin as well as host blood serum through the action of red blood cell transporters. As the parasite does not have any carbohydrate reserves (15, 16) or a tricarboxylic acid cycle (TCA) that is linked to glycolysis (17), it relies on glycolysis for free energy production from glucose (12), with an inherent low yield of two adenosine triphosphate (ATPs) per glucose. Hence, glucose uptake and lactate production by the infected RBC increases almost 100-fold (18), due to the high metabolic rate of the rapidly growing parasite.

The relative contributions of the different mechanisms to the development of elevated blood lactate and hypoglycemia are not well understood. Ideally, a whole-body description of glucose metabolism in the infected human host would allow us to gain quantitative insight into the disruption of glucose homeostasis. While some work has been done toward this, for example, modeling the cytoadherence and changes in the mechanical behavior of infected RBCs in blood flow (see *e.g.*, (19)), detailed models of metabolism at different levels of cells and organs in the human host are still lacking.

In previous studies, we highlighted our approach to constructing such a hierarchical model (20), and we started assembling the pieces by constructing and validating a detailed enzyme kinetic model of glycolysis in the *P. falciparum* trophozoite-stage parasite (21). In a subsequent study, a possible drug target was identified through model analysis and validated experimentally (22). In the current study, we construct and validate a model of a trophozoite-infected red blood cell, and expand it to the description of an infected red blood cell culture, including the different asexual blood stage forms. In addition, we perform an analysis of this model to identify a suitable drug target in the infected red blood cell and validate it experimentally. We show, through model simulation and experiment, that cytochalasin B has a similar inhibitory effect on the flux of the infected red blood cells (iRBCs) as it has on the flux of the isolated trophozoite, and that the glucose transporter is an effective target for the differential inhibition of flux in iRBCs over uninfected red blood cells (uRBCs).

## Results

### Model construction

For this study, a model of glycolysis in the parasite-infected RBC was required. We constructed the model using previously constructed and validated models for the parasite and the RBC.

Details of model construction are provided in the Model construction section in Experimental procedures. The kinetic model of *P*. *falciparum* glycolysis was constructed by Penkler *et al.* (21). The enzymes in the glycolytic pathway and glycerol branch of the parasite were kinetically characterized and the model validated using experimentally measured metabolite concentrations, metabolite fluxes (21), and a titration study of a glucose transport inhibitor for intact parasites (22). For the description of the metabolic activity of the RBC, the detailed enzyme mechanistic model of metabolism constructed by Mulquiney and Kuchel was used (23, 24, 25). This model includes glycolysis, the 2,3-Bisphosphoglyceric acid (2,3-BPG) shunt, the pentose phosphate pathway (PPP), the binding of metabolites to hemoglobin and Mg^2+^, and pH effects on reactions. The parasite and RBC models are available in the curated JWS Online Model repository (https://jjj.bio.vu.nl) (26, 27) as penkler1, vanniekerk1 (penkler1 model including competitive inhibition mechanism in the glucose transporter), and mulquiney.

In preparation for the inclusion of the parasite as a compartment inside the RBC, the units of the Mulquiney and Kuchel RBC model (M for metabolites and M/s for rates) were modified to be the same as those of the Penkler parasite model, that is, fmol/cell and fmol/cell/min, where cell indicates RBC in the case of the modified Mulquiney model, and parasite in the case of the Penkler model. The modified model was able to reproduce the results of the original Mulquiney model and is available on JWS Online as mulquiney1.

The Mulquiney and Kuchel model did not include a kinetic rate equation for the glucose transporter as the reaction was assumed to be in equilibrium. We considered it important to add a mechanistic equation for RBC glucose transport as it could play an important role in controlling the glycolytic flux of the iRBC. Here we employed a rate equation derived and parametrized by Potts and Kuchel (28). The model with the new glucose transport equation is available on JWS Online as mulquiney2.

**Figure 1:**
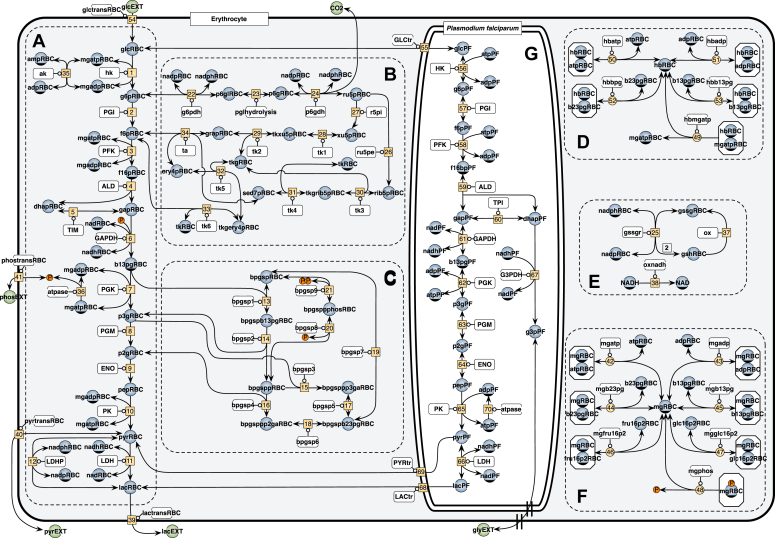
**The combined parasite and red blood cell model (dutoit1).***Orange blocks* show enzyme-catalyzed reactions, *blue circles* are the metabolites (each has an ODE), *green circles* represent the fixed metabolites for the steady state version of the model. *A*–*F*, the metabolic pathways in the red blood cell ((*A*) glycolysis; (*B*) PPP; (*C*) 2,3-BPG shunt; (*D*) hemoglobin binding; (*E*) glutathione oxidation and non-glycolytic NADH consumption; (*F*) magnesium binding) and (*G*) the glycolytic pathway in the parasite). Note the linking of the parasite metabolism to that of the red blood cell *via* the glucose transporter (GLCtr - reaction 55) and lactate and pyruvate transporters (LACtr – reaction 69; PYRtr – reaction 68).

Finally, the iRBC model (dutoit1) was created by linking the vanniekerk1 model with the mulquiney2 model through shared species in the RBC model that replace the fixed extracellular metabolites (glucose, lactate, and pyruvate) in the parasite model. A schema of this combined model (dutoit1) is shown in Figure 1.

This combined model, consisting of the *P. falciparum* and RBC models, describes the conversion of extracellular (to the RBC) glucose to end products lactate, pyruvate, and glycerol. Simulation results of the dutoit1 model can be compared to experimental data of flux measured in infected red blood cells. When extracellular metabolites in this model are unclamped, the time courses of these extracellular metabolites can be compared (dutoit2 model). Simulation outputs are in units of fmol/iRBC (metabolites) and fmol/min/iRBC (rates).

While the iRBC dutoit1 and dutoit2 models predict the metabolite dynamics of a single iRBC, an infected RBC culture model is required to predict the metabolite dynamics in a culture of iRBCs over a range of different levels of parasitemia. This model was constructed by including an additional uninfected red blood cell compartment (from mulquiney2) leading to a model consisting of iRBCs and uRBCs (dutoit3 model). See the Model construction section in Experimental procedures for further details. Model units are the same as for dutoit1 and dutoit2.

It is important to note that no further changes were made to the models when simulating experiments, apart from setting the extracellular environment, that is, the concentrations of metabolites external to the red blood cell compartment(s) (glucose = ConcGlcEXT, lactate = ConcLacEXT, glycerol = ConcGlyEXT, pyruvate = ConcPyrEXT, and phosphate = ConcPhosEXT), the volume of the incubation (or plasma) compartment (vBld), and the volume of the parasite (Vpf). For the dutoit3 model, we also included a parameter to change the parasitemia (par).

### Parasite volume using fluorescence microscopy

As the parasite volume is required by the model, and for the normalization of experimentally measured fluxes, we measured parasite volumes in a well-synchronized, infected culture treated with a cytosolic stain, using fluorescence microscopy.

**Figure 2:**
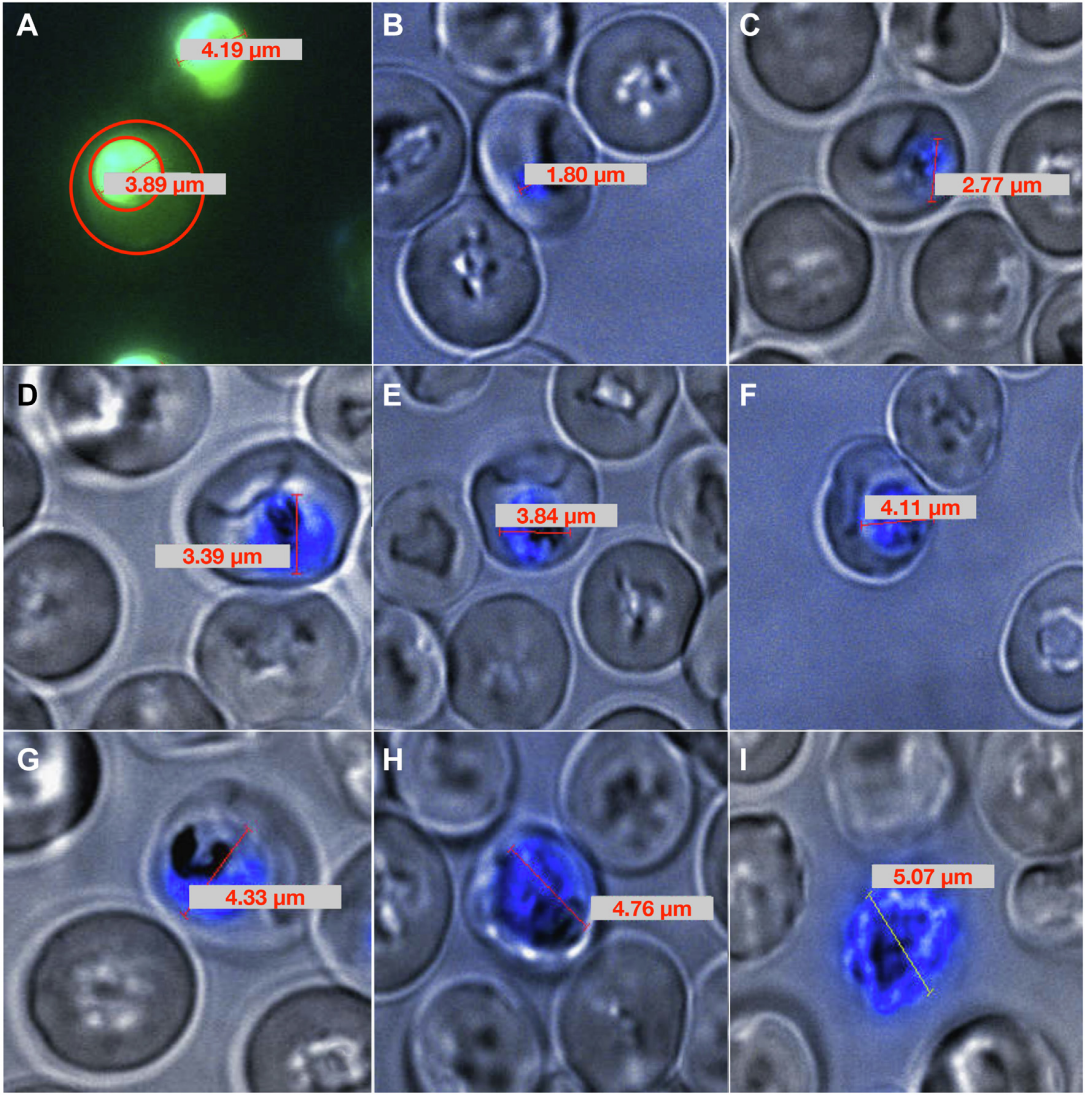
**Infected red blood cells visualized using fluorescence microscopy.** The outlines of the infected red blood cells can be distinguished by the difference in emission intensity of the cytosolic stain (*A*). Overlaid fluorescence emission (from Hoechst DNA stain) and transmission images of the infected red blood cells show the diameter of the intraerythrocytic parasite compartments (*B*–*I*). The pictures shown are representative of ring- (*B*), early trophozoite- (*C*), mid-late trophozoite- (*D*–*G*), and schizont-stage parasites (*H*–*I*). *Red lines* and accompanying values indicate diameter measurements.

Figure 2*A* shows that the outlines of the iRBCs can be distinguished by the difference in emission intensity of the cytosolic stain. Figure 2*B* - I show examples of measurements in a culture at specific time points to determine the diameter of the growing parasite inside the RBC. Diameters were then used to calculate parasite volume, assuming a spherical parasite cell. For the ring stage, the measurement was performed at 7 to 13 h post-invasion of the red blood cell, for the trophozoite stage at 32 to 35 h, and for the schizont stage at 42 to 48 h post-invasion.

The parasite volumes (mean ± SD) obtained in this manner were 5 ± 2 fl for the rings, 32 ± 8 fl for the trophozoites, and 74 ± 17 fl for the schizonts. These values correspond fairly well with the range of volumes reported in the literature: 4 fl (ring), 25 to 45 fl (trophozoite), and 70 to 74 (schizont) in the model of Lew (29) using a red blood cell volume of 90 fl (30), and 20.5 to 28 fl for the trophozoite determined using experimental measurements of intracellular water volume (31, 32).

### Enrichment of iRBCs in the trophozoite stage

**Figure 3:**
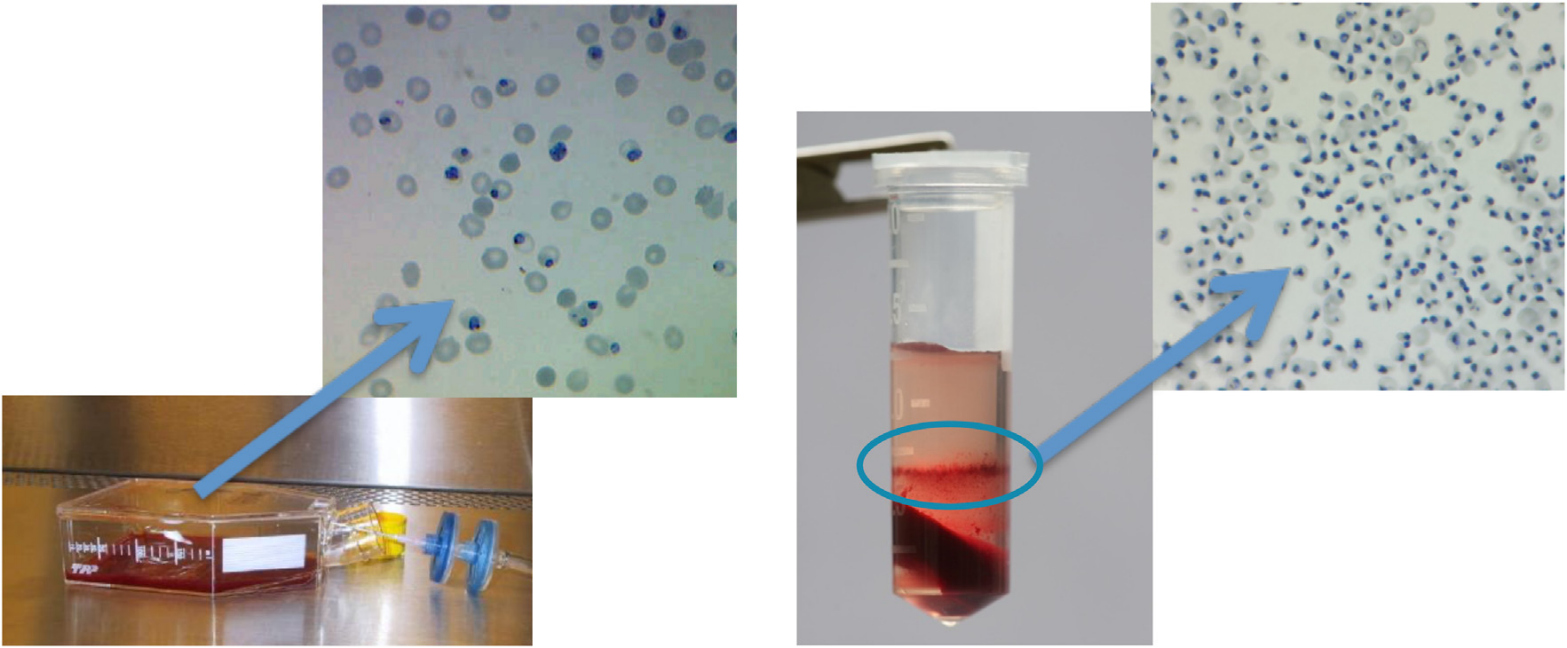
**High parasitaemias are obtained using the density gradient separation protocol.** Normal culturing protocol showing 15% parasitemia (*left*), whereas enrichment *via* density gradient separation produces parasitemia >90% (*right*). Cells were stained with DNA-based Giemsa stain.

To obtain a high percentage of iRBCs for subsequent flux measurements, trophozoite-stage iRBCs were enriched using a density gradient separation protocol. Whereas normal culturing protocols produce a parasitemia of <25% on average (with parasitemia being iRBCs as a percentage of all RBCs), this protocol consistently produces >90% parasitemia. Figure 3 shows parasitemia as is typical of normal culturing methods (left) as well as the interface of the two Percoll layers containing the iRBCs alongside a slide view of stained parasites showing the resultant high parasitemia from the enriched cells (right).

### Glycolytic flux determination and model validation

To validate the combined model, we compared the model-predicted consumption of glucose and production of lactate, and glycerol with experimentally measured rates. The model prediction of the steady-state fluxes was determined using the dutoit1 steady-state model, after setting the extracellular (to the iRBC) metabolites to fixed concentrations to mimic the validation experiment: 5 mM for glucose, 5.6 mM for phosphate, 0 mM for lactate, pyruvate, and glycerol, and vBld = 4050 fl. Additionally, we included uncertainty in parasite volume based on the time at which the cells are in the trophozoite stage (24 fl–40 fl at 32–35 h post-RBC invasion). The parasite volume is defined as a parameter in the model (Vpf).

We used the lactate production flux as a measure of glycolytic activity. We, therefore, incubated enriched trophozoite-stage iRBCs with glucose and measured the production of lactate external to the iRBCs enzymatically. This resulted in an experimental flux value (mean ± SEM) of 0.681 ± 0.038 fmol/min/iRBC (n = 6), which compared well with our dutoit1 model prediction of 0.419 to 0.695 fmol/min/iRBC for a trophozoite of volume 24 to 40 fl at 32 to 35 h post-invasion (and 0.557 fmol/min/iRBC for an average 32 fl trophozoite). For the same trophozoite volume range, the model also predicts the glucose consumption flux as 0.230 to 0.381 fmol/min/iRBC (experimental value of 0.399 fmol/min/iRBC) and the glycerol production flux as 0.0205 to 0.0337 fmol/min/iRBC (experimental value of 0.030 fmol/min/iRBC).

**Figure 4:**
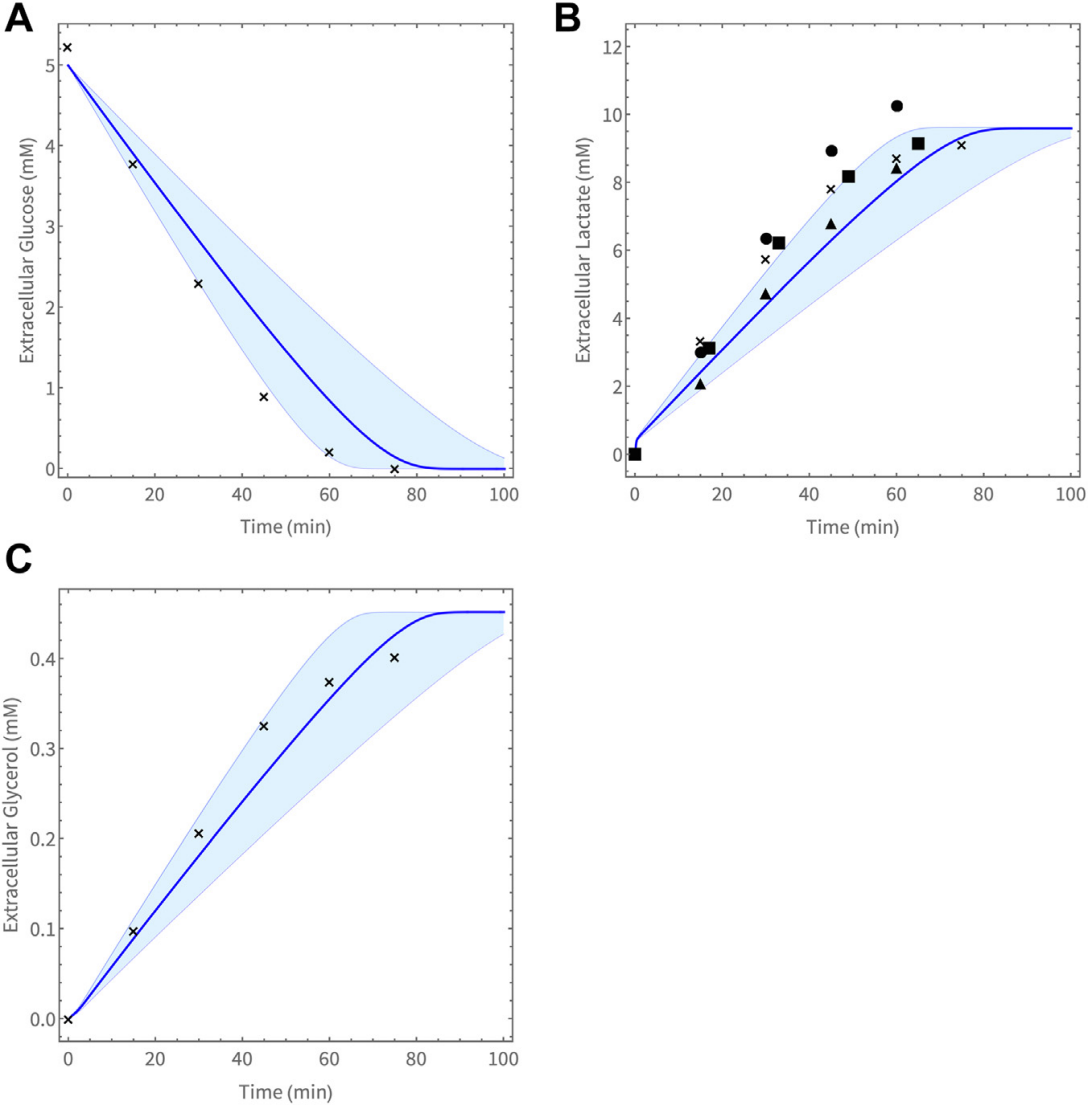
**Data and model prediction of glucose runout experiments.** Comparison of the model (dutoit2) predicted and experimentally measured values of the change in extracellular (to the iRBC) metabolites over time for the consumption of (*A*) glucose and production of (*B*) lactate and (*C*) glycerol. *Blue lines* with bands indicate the model’s predicted activity for the average measured trophozoite volume ± SD at 32 to 35 h post-invasion.

We simulated the time-dependent change in extracellular (to the iRBC) metabolites using the dutoit2 model and validated these simulations using the experimentally measured traces of the extracellular glucose, lactate, and glycerol (Fig. 4). Data from biological repeat experiments were standardized to an incubation volume (vBld in the model) of 4050 fl per cell.

### The effects of parasitemia and glucose on flux

**Figure 5:**
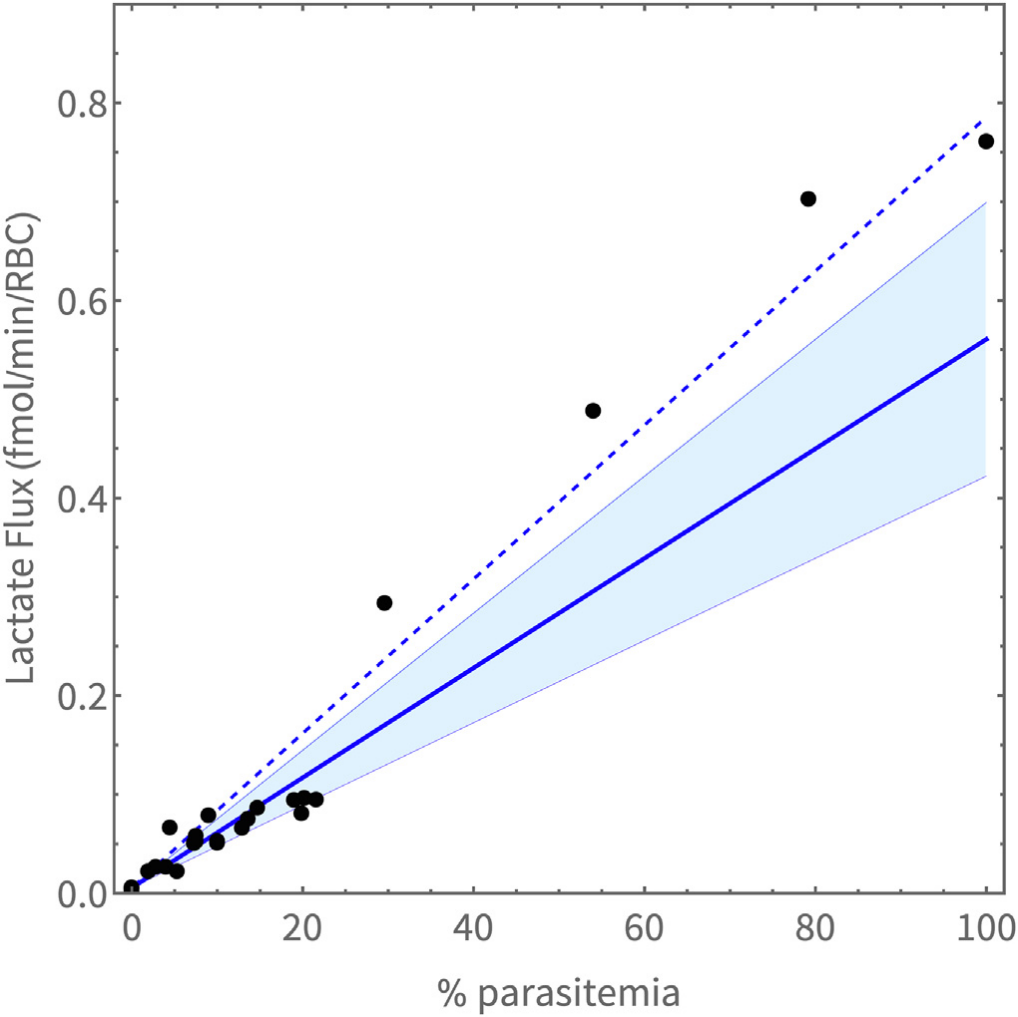
**Steady-state lactate flux as a function of parasitemia.** Steady-state lactate flux as a function of parasitemia determined for trophozoite-stage infected red blood cells in 5 mM glucose. For parasitemia of 30% and above, dilutions were made from the enriched culture (100%), and for lower values of parasitemia, cultures were grown to the % parasitemia shown. *Solid blue line* with bands indicates the dutoit3 model predicted activity for the average measured trophozoite volume ± SD at 32 to 35 h post-invasion (32 ± 8 fl). The *blue dashed line* indicates the model prediction when using the 45 fl volume of (29).

Lactate production was measured in a series of infected cultures with varying parasitemia, incubated in 5 mM glucose. Lactate flux as a function of parasitemia is shown in Figure 5, where the uRBC lactate flux (obtained from an experiment using uRBCs) serves as the 0% parasitemia point and the enriched iRBC lactate flux as the 100% parasitemia point. For parasitemias of 30% and above, dilutions were made from the enriched culture and for lower values of parasitemia, cultures were grown to the % parasitemia shown.

This experiment was simulated in the dutoit3 model to obtain a prediction of flux as a function of parasitemia. The effect of the variation in parasite volume (Vpf) is reflected in the blue band for parasites ranging between 24 and 40 fl when the culture is harvested at 32 to 35 h post-invasion for the flux experiment. Different parasitemias were simulated by varying the par parameter between 0 and 1.

As seen in Figure 5, the enriched cultures (parasitemia higher than 30%) exhibited a flux greater than that predicted by the model, whereas model predictions were in good agreement with results for cultures grown to sub 30% parasitemias. The reason for this is currently unclear although it is evident that the model prediction improves if the slightly larger volume of 45 fl (29) is used for Vpf.

**Figure 6:**
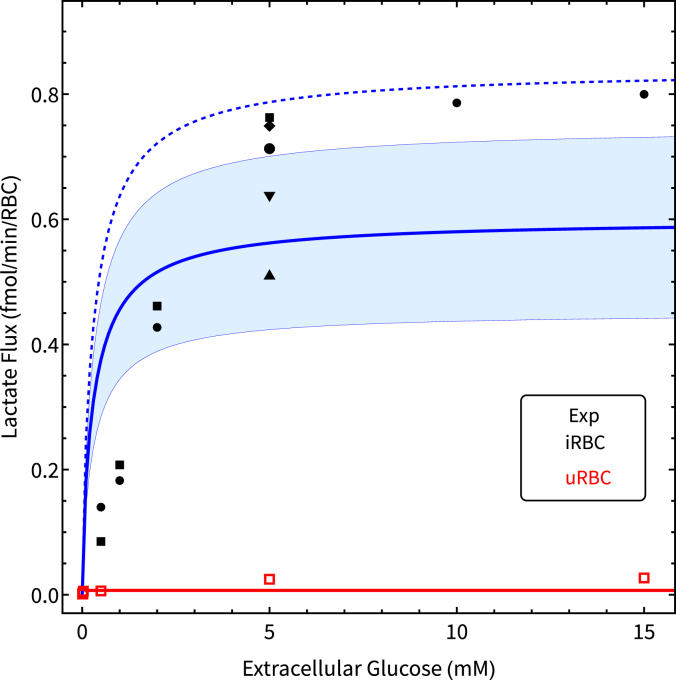
**Steady-state lactate fluxes as a function of extracellular glucose.** Steady-state lactate flux of 100% trophozoite iRBC incubations (*black symbols*) and uRBCs (*red symbols*) as a function of extracellular (to the RBCs) glucose, normalized to activity per cell. The model used was dutoit3 with either 0 (*red line*) or 100% parasitemia for the *solid blue line* with bands indicating the model prediction for the average measured trophozoite volume ± SD at 32 to 35 h post-invasion (32 ± 8 fl). The *blue dashed line* indicates the model prediction when using the 45 fl volume of (22).

To measure the effect of different extracellular (to the RBCs) glucose concentrations on the flux, 100% trophozoite-stage iRBCs, as well as uRBCs were incubated with different concentrations of extracellular glucose, and the lactate flux was determined for each case (Fig. 6).

This experiment was also simulated in the dutoit3 model at 0% or 100% parasitemia (setting the parameter par = 0 or 1) by varying the fixed extracellular (to the RBCs) glucose concentration. The effect of the variation in parasite volume (Vpf) is reflected in the blue band for parasites ranging between 24 and 40 fl when the culture is harvested at 32 to 35 h post-invasion for the flux experiment. The model simulation at 0% parasitemia is shown by the red line. The model prediction slightly overestimates the flux at low glucose and underestimates it at high glucose although the latter does improve when using the larger volume of 45 fl (29) as indicated by the dashed blue line.

### Comparison of different stage lactate fluxes

We measured the experimental lactate flux in enriched ring and schizont cultures and corrected for uRBC contributions. Lactate production was measured over a period of at least 30 min. From the lactate gradient over time and the number of cells (corrected for uRBCs), the flux per iRBC was calculated.

**Figure 7:**
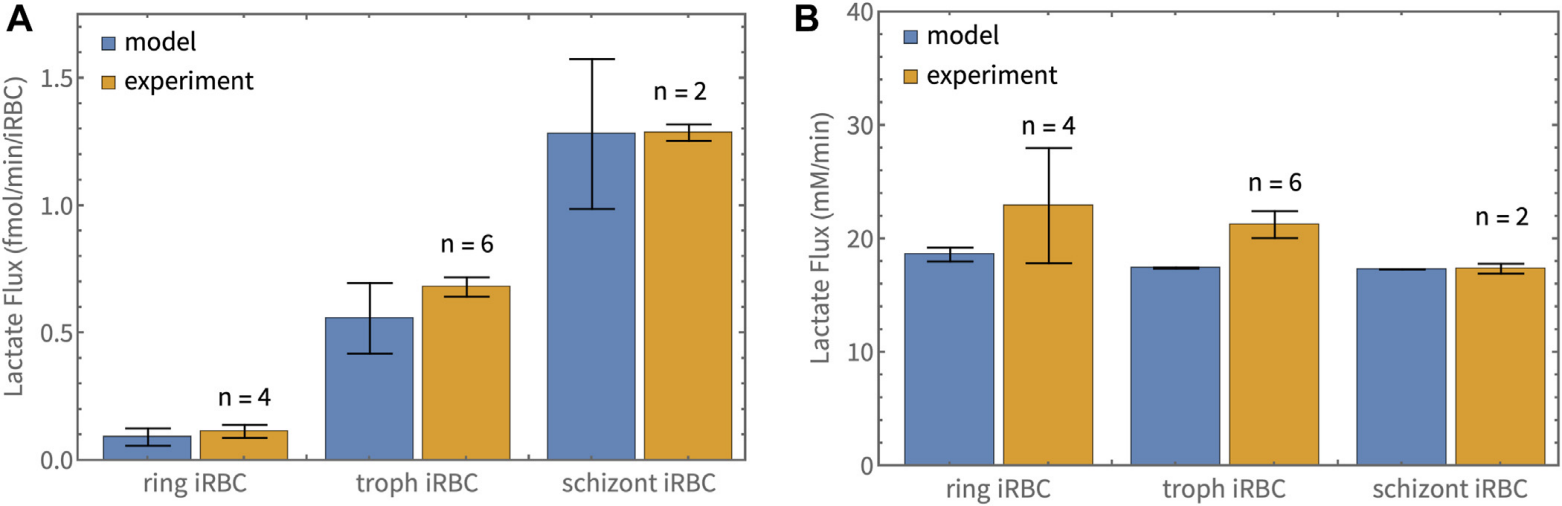
**Comparison of measured and predicted activity of different parasite stages.** Comparison of measured and predicted activity of different parasite stages. Model-predicted (*blue bars*) and experimentally measured fluxes (*orange bars*) of the iRBC are shown per iRBC (*A*), and normalized to the respective parasite volumes in the iRBC (*B*). Experimental results are shown as mean ± SEM. Bars for model predictions indicate the prediction using the mean volume of the parasite stage, with error bars showing the range obtained for the mean ± SD of the volume. The experimental results in (*B*) were normalized to the average volumes of the relevant parasite stages (5 fl ring, 32 fl trophozoite, 74 fl schizont).

For the ring stage (n = 4), we obtained a mean ± SEM of 0.115 ± 0.025 fmol/min/(ring iRBC), for the trophozoite (n = 6), we obtained 0.681 ± 0.038 fmol/min/(trophozoite iRBC), and for the schizont stage (n = 2), we obtained 1.29 ± 0.03 fmol/min/(schizont iRBC). Differences between these fluxes were determined to be statistically significant (Student’s *t* test, *p* < 0.001). These results are shown as the orange bars in Figure 7*A*.

For comparison of the activity per unit volume of the parasite, we normalized the total activity per iRBC to the average stage-specific parasite volume (5 fl for the ring stage, 32 fl for the trophozoite stage, and 74 fl for the schizont stage). The results are shown as the orange bars in Figure 7*B* (ring stage 22.9 ± 5.1 mM/min, trophozoite stage 21.3 ± 1.2 mM/min, and schizont stage 17.4 ± 0.4 mM/min). Differences between these normalized fluxes were determined to not be significant (Student’s *t* test, *p* > 0.05).

To obtain model predictions of glycolytic flux in the ring, trophozoite and schizont-stage iRBC, the experimentally measured volumes (see Parasite volume using fluorescence microscopy section) are used for the parasite volume parameter Vpf in the dutoit1 model (5 fl ring, 32 fl trophozoite, 74 fl schizont). Flux predictions using the average size parasite stages are shown as the blue bars in Figure 7. We also consider the effect of variability in parasite volumes (mean ± SD), resulting in a range of flux predictions depicted by the error bars on the blue bars. When taking variability in parasite volumes and experimental SEM into account, the model predicts the flux per iRBC as well as the flux per unit parasite volume with good accuracy for all parasite stages.

### Targeting the pathway flux

To identify reactions with substantial control on the lactate production flux of the infected red blood cell, we performed a metabolic control analysis (33, 34) on the dutoit1 model. These reactions could, in principle, serve as drug targets since inhibiting them would decrease the flux significantly, thereby reducing free energy production in the parasite. As was also seen in the case of the isolated parasite (22), the parasite GLCtr (glucose transporter), HK (hexokinase), PFK (phosphofructokinase), and GAPDH (glyceraldehyde 3-phosphate dehydrogenase) have the largest control on lactate flux with control coefficients of 0.29, 0.33, 0.29 and 0.10, respectively. Similar to the model analysis performed in (22), we then analyzed the model to explicitly determine the effect of competitive inhibitors on the pathway fluxes using inhibitor titrations. To quantify the differential effect that an inhibitor could have on the RBC *versus* the parasite, we modeled the effect of inhibitors on the glycolytic enzymes in the parasite and on RBC orthologs simultaneously. For each of the reactions, we simulated a generic competitive inhibitor for the parasite and RBC enzymes, by multiplying the relevant K_M_ values of the enzyme with a factor (1 + [I]/K_i_). Here we assumed the same [I]/K_i_ in both the parasite and the RBC. We then analyzed the extent of inhibition of both the enzyme activity and the pathway fluxes as a function of the inhibitor concentration in both compartments. RBC flux inhibition with respect to its own enzyme activities was tested in the iRBC (dutoit1) and uRBC (mulquiney2) models and similar results were obtained. Results presented hereafter specifically refer to the glycolytic flux in the RBC cytosol and the glycolytic flux in the parasite cytosol of the iRBC model.

**Figure 8:**
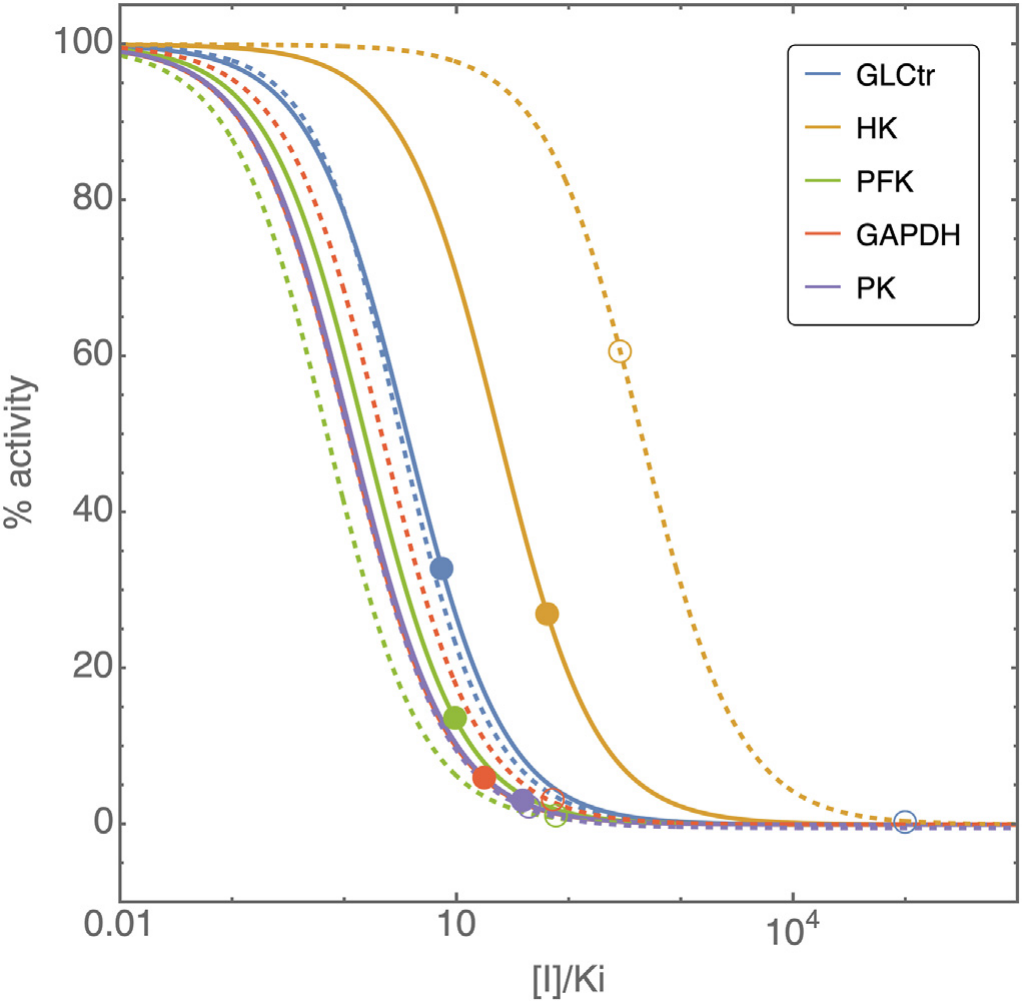
**Model simulations for competitive inhibitor titrations of selected glycolytic enzymes.** Solid and dashed curves indicate the activities of the parasite and red blood cell enzymes, respectively. Color is used to indicate orthologs. Activities are expressed as % value of uninhibited activity and plotted as a function of the inhibitor concentration (normalized to its K_i_ value). *Disks* and *circles* indicate the [I]/K_i_ values (x-axis) and the corresponding % activity of the enzyme (y-axis) where inhibition leads to a 50% inhibition of the glycolytic flux in the parasite and red blood cell respectively. Note that for 50% inhibition of flux in the parasite, the required [I]/K_i_ for a glucose transport inhibitor is ±10 (*blue disk*) whereas for the red blood cell, this value would be close to 10^5^ (*blue circle*).

In Figure 8 the inhibition of the activities of selected glycolytic enzymes are shown as a function of the inhibitor concentration. The enzymes chosen for this figure were shown in (22) to be those that required the lowest [I]/K_i_ to inhibit the parasite flux by more than 50%, namely, GLCtr, HK, PFK, GAPDH, and pyruvate kinase. Solid and dashed curves indicate the activities of the parasite and RBC enzymes respectively. Color is used to indicate orthologs. The points where inhibition leads to a 50% reduction in glycolytic fluxes (determined as the flux through hexokinase of either the parasite or the RBC) are indicated by a disk (parasite) and an open circle (RBC). In all cases, the parasite enzymes lead to 50% inhibition of flux at lower [I]/K_i_ values compared to their RBC orthologs (the disks are always to the left of the circles of the same color). The enzyme that requires the lowest [I]/K_i_ to inhibit the parasite flux by 50% (left-most disks) is the glucose transporter (GLCtr), followed by PFK, GAPDH, pyruvate kinase, and HK. In addition, the [I]/K_i_ required to inhibit flux by 50% is four orders of magnitude lower for the parasite GLCtr than for the RBC GLCtr.

**Figure 9:**
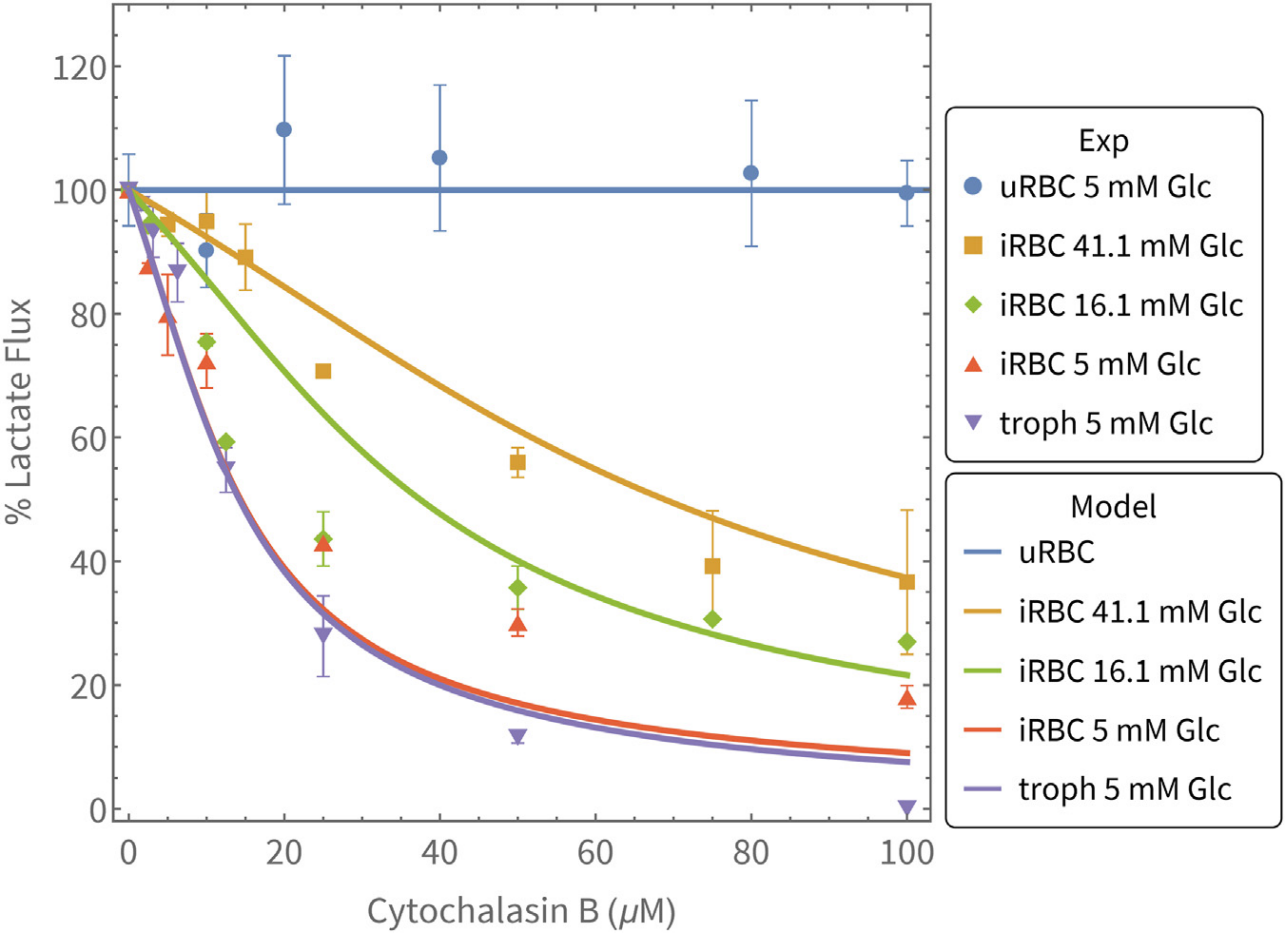
**Cytochalasin B inhibitor titrations of glycolytic flux in uninfected and infected red blood cells at different glucose concentrations.** Glycolytic flux was measured in terms of the lactate production rate at different cytochalasin B concentrations and is shown as a % of the uninhibited flux. Flux measurements were performed for uninfected (*blue*) and 100% enriched iRBCs at different glucose concentrations (*yellow*, *green*, and *orange*). The dutoit1 model predictions of the lactate production fluxes are indicated by the solid curves (*yellow*, *green*, and *orange*). The *blue line* indicates the 0% inhibition seen in the uRBC. For comparison, *purple markers* show the results of inhibition in the isolated trophozoite, and the *purple curve* the corresponding vanniekerk1 model simulation (22). Error bars are indicative of SEM where the measurement was performed in two or more biological repeats.

To test the effect of glucose transporter inhibition on glycolytic flux experimentally, we incubated uRBCs and 100% enriched trophozoite-stage iRBCs with varying concentrations of glucose and cytochalasin B and determined the steady-state lactate flux for each scenario. Results are shown in Figure 9 and indicate a significant inhibition of flux in the iRBC at 100 μM cytochalasin B (yellow, green, and orange data) that diminishes as glucose concentration increases. For comparison, the inhibition data for the isolated trophozoite (from (22)) is shown (purple data), indicating that the glycolytic flux in the iRBC is slightly less inhibited by cytochalasin B than that of the isolated parasite.

In the concentration range of 0 to 100 μM, it is evident that the flux in the uRBC is not significantly inhibited (blue data). We therefore proceeded to simulate the inhibition in the dutoit1 model which contains a mechanistic description of the effect of cytochalasin B on the glucose transporter of the parasite only (22). Simulation results are shown as curves in Figure 9. For these simulations, we set vBld = 2250, Vpf = 32, varied ConcGlcExt (the concentration of glucose external to the iRBC) for the different curves, and scanned cyt (the cytochalasin concentration) for the x-axis. The model was able to predict the experimentally observed flux inhibition with good accuracy.

## Discussion

Mathematical modeling can be a powerful tool to investigate the molecular origins of disease symptoms and to identify targets for treatment. To this end, sufficiently detailed models of a disease system must be constructed with parametrization based on experimental data and validated using independent experiments.

In this study, we used a bottom-up approach that relies on combining detailed enzyme kinetic models of individual biological systems into a multi-compartment model. This builds on previous work where we constructed, validated, and analyzed a detailed enzyme kinetic model of glycolysis in the trophozoite-stage of the *P. falciparum* malaria parasite (21, 22). Here, we created a detailed enzyme kinetic model of glycolysis in a trophozoite-infected red blood cell by combining the parasite model with an existing model of metabolism in the human red blood cell (23, 24, 25).

Model construction entailed converting the units of the original RBC model by Mulquiney & Kuchel to be compatible with the units of the Penkler parasite model. We added a rate equation for the glucose transporter of the RBC (which was absent in the model previously) as derived and parametrized by Potts and Kuchel (28). Finally, the combined model was created by linking the extracellular metabolites in the parasite model to the same (intracellular) metabolites in the RBC.

First, we determined the volumes of the ring, trophozoite, and schizont stages using fluorescence microscopy as these would be required to set the parasite volume in the model and to determine the glycolytic flux per unit parasite volume for comparison of the different stage fluxes. The volumes agreed well with the values from the literature.

The trophozoite-stage iRBC model was validated experimentally with whole-cell glycolytic flux measurements performed at varying degrees of parasitemia and extracellular (to the RBCs) glucose concentration. Model predictions mostly compared well to experimental results given uncertainty in the volume of intra-erythrocytic parasites and the fact that no model parameters were refitted to experimental data.

To adapt the kinetic model of the trophozoite-stage iRBC to describe the other stages of the intra-erythrocytic phase, we assumed non-differential glycolytic enzyme expression in the different stages. This allowed us to simply replace the parasite volume in the model with the volume of the relevant stage (ring, trophozoite, or schizont). In principle, one could measure the activity of the glycolytic enzymes in all stages but some experimental challenges exist: due to the fact that rings are small, one would need a large volume of culture, which is labor and cost-intensive, and due to the multi-nucleated nature of schizonts they are structurally fragile and difficult to isolate. Trophozoites seem to offer a good middle ground by allowing for easy isolation and a sufficient amount of biomass.

To test our assumption of non-differential glycolytic enzyme expression, we determined the glycolytic fluxes of the ring iRBC and schizont iRBC and found that there were no statistically significant differences between the stage-specific fluxes once normalized to cell volume (mM/min). Volume-adapted model predictions of the fluxes were very similar to experimental results, further validating our assumption and the accuracy of the model. Studies have shown that there are changes in mRNA expression and protein abundance levels throughout the 48 h of the intra-erythrocytic phase (35, 36), however, when considering proteins that function in the same pathways, their abundances seem to be coordinated (36). It might also be the case that whatever relative changes there are amongst the glycolytic enzymes, these do not result in an overall noticeable effect on the steady-state fluxes. We, therefore, conclude that if there is differential expression of glycolytic enzymes between the different stages, this apparently does not lead to a noticeable effect on specific flux, which we observed to be the same for the different stages tested. The appearance of an increasing glycolytic flux as the parasite grows is therefore a result of an increase in parasite biomass leading to a larger flux per cell (fmol/min/iRBC).

Evident from the experimental results of Figures 5 and 7 is the dramatic increase in the lactate flux of the iRBC compared to the uRBC. At 5 mM extracellular glucose, the uRBC flux was determined to be 0.0062 ± 0.0011 fmol/min/RBC (n = 4), whereas the flux of the trophozoite iRBC was 0.681 ± 0.038 fmol/min/iRBC (n = 6). This agrees also with model predictions of 0.00600 fmol/min/RBC (mulquiney) and 0.557 fmol/min/iRBC (dutoit1), and reflects the approximately 100-fold increase stated in (18).

Subsequently, the model was analyzed to identify good targets for inhibiting the glycolytic flux of iRBCs through a competitive inhibitor acting on both parasite and RBC enzymes. The analysis showed that inhibition of some parasite enzymes leads to significant inhibition of lactate flux at a much lower inhibitor concentration compared to that required to affect orthologous enzymes in the RBC (assuming the same binding constant). This not only points to good targets for the inhibition of lactate flux but also to targets that could be used to differentially affect the parasite more severely than the RBC. As was the case in (22), the parasite glucose transporter was identified as the reaction for which inhibition has the strongest effect on lactate flux.

Cytochalasin B was used for experimental characterization of the effect of glucose transport inhibition on lactate flux. In the case of uRBCs, no inhibition of flux was observed. There is evidence that cytochalasin B binds to the GLUT1 glucose transporters in the RBC membrane (37, 38). However, due to the overcapacity of the transporters (requiring in excess of 99% inhibition to affect flux (37)) no effect was observed in the range of concentrations tested here.

For the iRBC, the model was able to predict the inhibition as a function of cytochalasin concentration adequately (and with no parameters refitted). However, while the model prediction of inhibition in the iRBC at 5 mM glucose appears similar to the prediction for the isolated parasite shown in (22), experimental results show a stronger inhibition of flux in the isolated parasite compared to the iRBC (purple data compared to orange). It, therefore, seems that less inhibitor reaches the parasite inside the RBC compared to the isolated parasite. In the case of the iRBC, cytochalasin must cross two barriers to reach the parasite, namely, the membrane of the RBC and the parasitiphorous vacuolar membrane (PVM). Cytochalasin B has also been shown to bind to F-actin filaments (39, 40) which can restrict the amount of inhibitor that reaches the parasite glucose transporter and in so doing, lead to the incomplete inhibition of the glycolytic flux in the iRBC.

In summary, here we reported in a transparent and reproducible manner, the construction and validation of an infected red blood cell model. Importantly, no parameters in the model were refitted to new experimental data. We also showed how model analysis can be used to identify effective targets for inhibition in the combined system and validated a target using further experiments.

The combined RBC-parasite model will form an integral part of a hierarchical model for the description of glucose metabolism in a malaria-infected human host. Whereas a complete model for the disease at the whole-body level would have to describe all the disease mechanisms (associated with parasite metabolism, and disruption of host metabolism due to physical obstruction and immune response), the next step would be to quantify the contribution of the parasites’ metabolism by constructing a model that incorporates its effect on whole-body glucose metabolism, as we conceptually demonstrated in (20). The combined RBC-parasite model forms one part of such a model, the other part being a whole-body model that can accurately describe lactic acidosis and hypoglycemia. Currently, very few appropriate models exist, although there have been some recent developments, for example (41). In the end, a more complete model of the disease state should be sufficiently detailed to gain a mechanistic understanding of the origin of metabolic pathologies of malaria infection. More importantly, perhaps, it should also enable the type of drug target analysis we demonstrated here, which would allow one to investigate the effect that targeting reactions in the parasite has on the symptoms at the level of the whole body.

## Experimental procedures

### Culturing and synchronization of *P. falciparum* cultures

*P. falciparum* (D10 strain) was cultured in a standard 250 ml airtight culturing flask containing: 3 to 4% hematocrit, a trimix gas mixture consisting of 3% oxygen, 4% carbon dioxide, and 93% nitrogen, incubated at 37 °C in complete culturing medium. All reagents were obtained from Sigma-Aldrich unless stated otherwise. The complete culturing medium consists of RPMI-1640 (R6504, 10.4 g/l) culture medium supplemented with 0.5% m/v Albumax II (R), 22.2 mM glucose (33.3 mM final glucose concentration), 25 mM HEPES, 3 mM hypoxanthine, 25 mM sodium bicarbonate and 50 μg/ml gentamycin sulfate as described previously (21, 42, 43, 44, 45).

Blood (A^+^) was obtained from the Western Cape Blood Service (from anonymous donors), in standard blood transfusion bags containing 20 to 30 ml of anticoagulant citrate dextrose solution (ACD-A) per 450 ml of blood. Blood was transferred to 50 ml conical tubes in a sterile environment. Tubes were filled to 50% blood and 50% culture medium and inverted to mix well. Tubes were centrifuged (1300*g*) after which the supernatant was aspirated and the cells resuspended to 50 ml in fresh medium. This was repeated for two cycles. Washed RBCs were stored in a complete culturing medium at 4 °C until use.

The culture medium was replaced daily by centrifugation of the iRBC culture (750*g*, 3 min), decanting of the spent medium, and resuspension to 50 ml with fresh medium. RBCs were added to an iRBC culture when at the trophozoite stage to maintain a 3 to 4% hematocrit. Unless a high parasitemia was desirable, cultures were split when parasitemia reached 5% or higher, by dividing a 50 ml culture into two 50 ml conical tubes and filling up each with culture medium and the appropriate volume of RBCs.

The cultures were synchronized by suspending centrifuged iRBCs (750*g*, 3 min), in a prewarmed, sterile 5% sorbitol solution in distilled water (dH_2_O) and incubating the suspension (with occasional mixing) at 37 °C for 5 to 10 min (46, 47). After further centrifugation, the sorbitol solution was aspirated, and the culture was washed once in fresh culture medium before being cultured as described above. Sorbitol treatment leaves uninfected, as well as ring-infected RBCs intact, but lyses the trophozoite and schizont-stage iRBCs. RBCs infected with early trophozoites (late rings) will also survive synchronization, thus the timing of the sorbitol treatment is crucial to achieving tightly synchronized cultures. Cultures were synchronized 1 to 2 times a week on average. When the parasites were in the trophozoite stage, the cultures were kept overnight on an orbital shaker (20 RPM, 37 °C) in order to maximize reinvasion efficiency and minimize multiply-infected RBCs (48).

### Fluorescence microscopy

For the volume measurements of infected red blood cells, a well-synchronized, infected culture at low parasitemia was kept at standard culturing conditions for at least 24 h before the measurements took place. An aliquot of the cell suspension was centrifuged on a desktop centrifuge (6500*g*, 3 min). A volume of 2.5 μl of the pelleted cells was then resuspended in 2 ml of culture medium. To this suspension, 0.25 μl of a cytosolic stain (CellTracker Green CMFDA; Life Technologies) was added (1:4000 dilution; 125 μM final concentration) and incubated for 45 min at 37 °C. After incubation, the suspension was pelleted (650*g*, 3 min), the supernatant removed, and cells resuspended in 2 ml of culture medium. A volume of 10 μl of a nucleic acid stain (Hoechst 33342; Life Technologies) was added and incubated for 30 min at 37 °C. An aliquot of 50 μl was then taken and visualized on a fluorescent microscope (details below) to distinguish between the different compartments as the cytosol of the parasite has a greater emission than the cytosol of the red blood cell. The intensity of the cytosolic stain could therefore be used as an additional cross-check to ensure the identification of an infected red blood cell after visualizing the cells using normal light transmission microscopy.

The transmission image, together with the fluorescence image, was used to measure the diameter of the parasite. To calculate the volume, the diameter was used in the equation for the volume of a sphere, as the parasite is mostly spherical in shape in all three stages. Cells were visualized using a temperature-controlled Olympus Cell system, attached to an IX-81 inverted fluorescence microscope. It is equipped with an F-view-II cooled CCD camera and a 150 W Xenon lamp as light source, which is part of the MT20 excitation source. The Olympus UPlan Apo N 60×/1.4 Oil objective was used with a YFP filter set.

### Enrichment of infected RBCs in the trophozoite stage

Trophozoite-stage iRBCs were enriched using a density gradient separation protocol, a modification of the method described in (49, 50). This method involves the preparation of two Percoll solutions differing in density. The solutions are made up of an amount of Percoll, dH_2_O, and a 10× concentrated solution (containing RPMI, glucose, and HEPES, pH 7.2). For instance, an 85% Percoll solution would consist of 4.25 ml Percoll, 0.5 ml 10× solution, and 0.25 ml dH_2_O. A 60% Percoll solution would consist of 3 ml Percoll, 0.5 ml 10× solution, and 1.5 ml dH_2_O. Alanine (3% *w/v* final conc.) was added to both solutions. In a 2-ml Eppendorf tube, 500 μl of the 60% solution was layered on top of 500 μl of the 85% solution. This must be done very carefully so as not to disturb the interface between the layers. On top of the Percoll layers, 200 to 250 μl of packed iRBCs (collected at 750*g*, 3 min) is then centrifuged immediately in a benchtop centrifuge (9600*g*, 20 min, zero deceleration).

The layer of cells between the 85% and 60% Percoll layers contains the enriched trophozoite-stage iRBCs (see Figure 3 in the Results section). This layer is removed from each tube, pooled, and washed with pre-warmed (37 °C) 5× phosphate-buffered saline and spun (600*g*, 3 min). The cells were resuspended in approximately 10 ml of complete culturing medium per enriched Eppendorf tube for 30 min at 37 °C to allow the remaining alanine to equilibrate. The suspension was spun (600*g*, 5 min) to produce a pellet of infected cells, which can then be used for experimentation or put back into culture. *When the cells were subsequently cultured, this protocol was performed in a sterile environment.*

### Enrichment of infected RBCs in the ring stage

Early-stage parasites (rings) were enriched using a saponin hemolysis protocol (51). The saponin powder used contains 11.1% active component sapogenin, and thus the working solution (0.015% *w/v* and 0.001665% sapogenin) *was* prepared using 15 mg saponin powder in 100 ml Krebs/Ringer phosphate buffer containing (mM): NaCl, 68; Na_2_HPO_4_, 50; KCl, 4.8; MgSO_4_, 1.2 at pH 7.4. The solution was sterilized by filtration using a 0.2 μm pore size membrane filter.

Aliquots of *P. falciparum* culture with a parasitemia of 10% or higher were synchronized using sorbitol as described above, pooled, and centrifuged for 10 min (750*g*) into an approximately 3 ml cell pellet. The cells were then treated with 28 ml of cold 0.015% (*w/v*) saponin solution and immediately mixed by hand. The suspension was aliquoted into four tubes, each containing 7 ml, and incubated on ice for 30 min with occasional mixing. After centrifugation (5 min, 750*g*), the pellets were pooled and resuspended in 7 ml complete culture medium. After additional centrifugation (5 min, 750*g*), the supernatant was discarded, and the pellet was again resuspended in 5 ml 0.015% *w/v* saponin solution and incubated for 5 min on ice. Lastly, the suspension was centrifuged (5 min, 750*g*) and the cell pellet was washed three times with 7 ml complete culture medium each time. This protocol produces approximately 65% parasitemia with ±15% multiply-infected RBCs. Centrifugation steps during this procedure were done at 4 °C.

### Enrichment of parasites in the schizont stage

Schizont-infected red blood cells were obtained by enriching trophozoite-infected RBCs, as described above, which were then put into a 10 ml complete culturing medium, gassed as normal, and incubated for 13 h at 37 °C to reach the schizont stage. The aliquot was then pelleted (600*g*, 3 min on a desktop centrifuge) and gently resuspended in 50 ml normal culture medium for 30 min, pelleted again (600*g*, 3 min on a desktop centrifuge) and used in incubation for flux determination.

### Glycolytic flux measurements and whole-cell inhibition

Trophozoite-stage iRBCs were enriched using the density gradient separation method to produce nearly 100%-infected red blood cells. The metabolic activity of iRBCs was determined by incubating enriched (near 100% infected), intact iRBCs with glucose and measuring enzymatically the lactate produced and glucose consumed over time. The incubation consisted of packed iRBCs (750*g*, 3 min), in an RPMI (R1383, 8.4 g/l) and HEPES (50 mM) buffer, with the glucose concentration of interest, in a 1:15 to 1:20 ratio of cells:(incubation buffer). In the case of determining the effect of an inhibitor on lactate production, cytochalasin B (dissolved in DMSO, final concentration <0.5% m/v) was added to the incubation buffer in varying concentrations up to 100 μM. The control incubation contained no inhibitor, but an equivalent amount of DMSO.

Where lactate flux was determined as a function of parasitemia or glucose, the enriched cells were used in incubation as is, or diluted with uRBCs to a range of different final parasitemias of 30% and above. For parasitemia below 30%, cultures were grown to the relevant parasitemia. The incubations consisted of sedimented (750*g*, 3 min) cells in glucose, RPMI (8.4 g/l), and HEPES (50 mM) buffer in a 1:10 to 1:20 ratio of cells:(incubation buffer).

The metabolic activity of the ring- and schizont-infected RBCs were determined by incubating enriched iRBCs with 5 mM extracellular glucose and measuring enzymatically the extracellular lactate production over time. The incubation consisted of pelleted (750*g*, 3 min) iRBCs in glucose (5 mM), RPMI-R1640 (R1383, 8.4 g/l), and HEPES (50 mM) buffer, in a cell:(incubation buffer) ratio of 1:10 to 1:15 for rings and a 1:30 to 1:35 ratio for schizonts. In the case where the enrichment did not result in a near-100% parasitemia, the glycolytic activity of the ring-infected and schizont-infected RBC incubations were corrected with the flux of uRBCs. For this, uRBCs were suspended in the abovementioned incubation buffer and the extracellular lactate production was measured over time.

The cells from all incubations excluded 0.04% *v/v* trypan blue in their incubation buffer, as an indication of intact membrane integrity. A 10 μl sample was taken for cell counting using the Improved Neubauer hemocytometer. Time point samples were collected and centrifuged (800*g*, 1 min) and the supernatant was kept for enzymatic determination.

### Determination of extracellular metabolites

Extracellular L-lactate contained in the supernatant samples was quantified using an LDH and NAD^+^ linked assay. Standards and samples were incubated in an L-LDH (11 U/ml), NAD^+^ (1.6 mM), hydrazine (16 μl/ml), and HEPES assay buffer (150 mM HEPES; pH 7.6), incubated for 90 min at 21 °C, and absorbance measured at 340 nm (Varioscan microplate reader Thermo Electron Corporation). Hydrazine was added to prevent pyruvate from inhibiting LDH.

Extracellular glucose was determined using an HK/G6PDH and NADP^+^ linked assay. Standards and samples were incubated in an HK/G6PDH (3.4 U/ml HK, 1.7 U/ml G6PDH), NADP^+^ (5 mM), and HEPES assay buffer (150 mM HEPES, with sufficient Mg2^+^ in the form of MgSO_4_.7H_2_O (15 mM); pH 7.6), incubated for 30 min at 21 °C and absorbance measured at 340 nm (VarioSkan microplate reader, Thermo Electron Corporation).

Glycerol was measured using the method developed by Eggstein and Kuhlmann (52). Glycerol samples and standards were incubated in assay buffer (150 mM HEPES; pH 7.6, with MgSO_4_.7H_2_O (15 mM)) with glycerol kinase (8.4 U/ml), PEP (1 mM), NADH (0.4 mM), ATP (1 mM), pyruvate kinase (3 U/ml) and lactate dehydrogenase (3.6 U/ml). After 10 min at 21 °C, the oxidation of NADH was measured at 340 nm.

### Model construction

Model construction and analysis were performed using Wolfram Mathematica 13 (Wolfram Research, Inc).

The kinetics of the original parasite model (penkler1) in the trophozoite stage was defined in a cell water volume of 28 fl (measured at 21–30 fl (52)), from trophozoites harvested at 32 h post-invasion, resulting in a cytosol to protein ratio of 4.67 μl cytosol/mg total protein. In this work, all trophozoite-stage cells were harvested at 32 to 35 h post-invasion, resulting in a slightly bigger average volume of 32 fl.

To include the parasite as a compartment inside the RBC, the units of the two models (Penkler model for parasite, and Mulquiney and Kuchel model for RBC) were made compatible. The Penkler model (vanniekerk1) has a rate unit of fmol/min/parasite and a substance unit of fmol/parasite. In the rate equations, metabolites are divided by their compartment volumes (in fl), and the dissociation constants (K_M_ values) are given in M. V_max_ values are in M/min and are multiplied by compartment volumes to obtain rates in fmol/min/parasite. The Mulquiney model has a rate unit of M/s and a substance unit of M, with V_max_ values in M/s and K_M_ values in M. The units of the Mulquiney model were therefore changed to fmol/min/cell (rates) and fmol/cell (substances). This was accomplished by including a time-scaling factor in the rates (×60), explicitly dividing metabolites (which would now be in fmol/cell) by their compartment volumes (in fl) in the rates, and multiplying the rates by the relevant compartment volumes: Vrbc = 90 fl (measured at 82–91 fl (53)) for internal metabolites and vBld = 128.5 fl for extracellular metabolites. The extracellular volume (vBld) was set to reproduce the volume ratio reflected in the original model and is used to reproduce its results. This model is available as mulquiney1 on JWS Online.

The equation for glucose transport from Potts and Kuchel (28) was subsequently incorporated into the mulquiney1 model. It is based on experimental observations and considers the binding of the two different anomers of glucose to the integral membrane protein which transports glucose in and out of the RBC (with 36% of the equilibrium mixture for D-glucose in the *α* anomer and 64% in the *β* anomer form). Here we assumed that 0.36 of the blood glucose goes *via* the transport route for *α*-glucose while the other 0.64 is transported *via* the *β*-glucose route with a different binding affinity. Additionally, it was assumed that these two routes are reversible and symmetrical, with the use of Equations 1 and 2 in (28) the following rate equations were obtained for the two anomers:

$$v_{\alpha }=\frac{V_{\max\alpha }\cdot \alpha_{o}}{K_{\alpha }+\frac{K_{\alpha }}{K_{\beta }}\beta_{o}+\alpha_{o}}-\frac{V_{\max\alpha }\cdot \alpha_{i}}{K_{\alpha }+\frac{K_{\alpha }}{K_{\beta }}\beta_{i}+\alpha_{i}}$$

$$v_{\beta }=\frac{V_{\max\beta }\cdot \beta_{o}}{K_{\beta }+\frac{K_{\beta }}{K_{\alpha }}\alpha_{o}+\beta_{o}}-\frac{V_{\max\beta }\cdot \beta_{i}}{K_{\beta }+\frac{K_{\beta }}{K_{\alpha }}\alpha_{i}+\beta_{i}}$$

so that

$$v_{GLCtr}=v_{\alpha }+v_{\beta }$$

Here *α* and *β* denote the glucose anomers (*α* = 0.32[glc] and *β* = 0.64[glc]), subscripts *o* and *i* indicate extracellular and intracellular glucose respectively, and *K*_*α*_ = 0.0083 M and *K*_*β*_ = 0.007 M at 37 °C (29). The *V*_max_ values were assumed to be the same for both anomers and was determined by Potts and Kuchel to be 33 mmol/s/l of RBCs. Since the *V*_max_ values in the Mulquiney and Kuchel models (mulquiney and mulquiney1) are specified in M/s, this transporter’s *V*_max_ values were converted to the same unit:

$$V_{\max}=\frac{33\;mmol}{s\cdot L\;of\;RBC}=\overset{V_{\max}}{\overset{︷}{\frac{33\;mmol}{s\cdot L\;of\;RBCs}}}\cdot \overset{1/cytosolic\;ratio\;of\;RBCs}{\overset{︷}{\frac{1L\;of\;RBCs}{0.7\;L\;cytosol\cdot 1000}}}=0.047\;M/s$$

where the cytosolic ratio of 0.7 is the cell water fraction parameter as specified in the Mulquiney and Kuchel model. The model with the new glucose transport equation (given by the sum of $v_{\alpha }$ and $v_{\beta }$) is available on JWS Online as mulquiney2.

The iRBC model (dutoit1) was created by linking the vanniekerk1 model with the mulquiney2 model through shared species in the red blood cell model that replace the fixed extracellular metabolites (glucose, lactate, and pyruvate) in the parasite model. In each of the rate equations of the parasite, its metabolites are divided by the parasite cytosolic volume, Vpf (trophozoite stage), whereas the extracellular parasite metabolites (now also those internal to the RBC: glucose, lactate, and pyruvate) including all other RBC metabolites, are divided by the RBC compartment volume (Vrbc). External to the RBC, *i.e.*, in the blood plasma compartment, metabolites glucose, lactate, glycerol, pyruvate and phosphate are divided by the blood volume (vBld).

To investigate glucose runout and product formation, a derivative of the dutoit1 model was created where the extracellular (to the iRBC) metabolites are unclamped. To simulate this closed system with variable extracellular metabolites, additional equations for the extracellular metabolites glucose, lactate, pyruvate, phosphate and glycerol, were added to the current set of ODEs This model is available on JWS Online as dutoit2.

**Figure 10:**
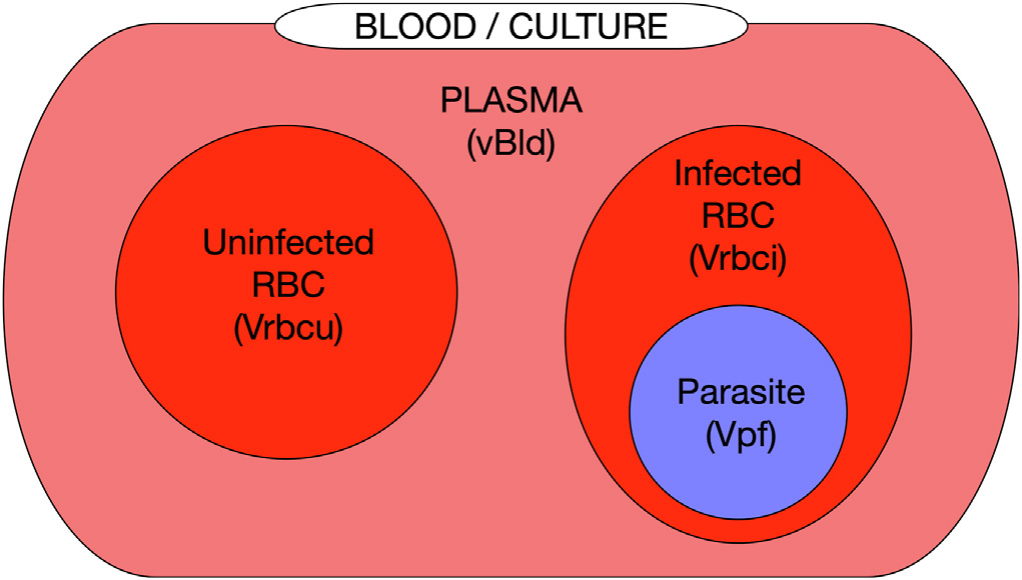
**Scheme showing the different volume compartments in the dutoit3 model.** The blood plasma/culture volume vBld, the uninfected RBC volume Vrbcu, the infected RBC volume Vrbci, and the parasite volume Vpf. Each volume compartment can consist of multiple cells but is simulated as a total volume.

To investigate the behavior of iRBC cultures of varying parasitemia, a model was created that consists of the dutoit1 model for the iRBCs and the mulquiney2 model for the uRBCs (Fig. 10).

In this model, the volume of the infected RBC compartment (Vrbci) is calculated by using the parasitemia (par) to determine the infected fraction of the total RBC volume. The uninfected RBC volume (Vrbcu) makes up the remainder. The parasite volume (Vpf) depends on Vrbci and on the ratio of volumes of one trophozoite parasite to one red blood cell (trophToRBC), for example 32/90 if the average size trophozoite is considered:

Vrbci=par⋅Vrbc

Vrbcu=Vrbc−Vrbci

Vpf=trophToRBC⋅Vrbci

## Data and model availability

Models, data, and Mathematica notebooks are available in FAIRDOMhub DOI: 10.15490/fairdomhub.1.investigation.604.1

Models are also available on JWS Online.

Mulquiney models: https://jjj.bio.vu.nl/models/?id=mulquiney

Penkler models: https://jjj.bio.vu.nl/models/?id=penkler

Du Toit models:

dutoit1: https://jjj.bio.vu.nl/models/dutoit1/simulate

dutoit2: https://jjj.bio.vu.nl/models/dutoit2/simulate/

dutoit3: https://jjj.bio.vu.nl/models/dutoit3/simulate/

dutoit1-user-88@jjj.bio.vu.nl

A SED-ML script for Figure 4 is available: https://jjj.bio.vu.nl/models/experiments/vanniekerk2023_fig4/

## Conflict of interest

The authors declare that they have no conflicts of interest with the contents of this article.
